# Plasma and Muscle Pharmacokinetics of Ceftriaxone in Nile Tilapia (*Oreochromis niloticus*) After Different Administration Routes

**DOI:** 10.3390/antibiotics14121253

**Published:** 2025-12-11

**Authors:** Pedro Marín, Orhan Corum, Duygu Durna Corum, Elena Badillo, María Teresa Yuste, Onder Yildirim, Ertugrul Terzi, Ruby C. Gonzales, Dan M. Arriesgado, Victor R. Navarro, Kamil Uney

**Affiliations:** 1 Department of Pharmacology, Faculty of Veterinary Medicine, University of Murcia, 30100 Murcia, Spain; pmarin@um.es (P.M.); mariateresa.yuste1@um.es (M.T.Y.); 2 Department of Pharmacology and Toxicology, Faculty of Veterinary Medicine, University of Hatay Mustafa Kemal, Hatay 31060, Türkiye; ddurnacorum@gmail.com; 3 Department of Aquaculture, Faculty of Fisheries, Mugla Sıtkı Kocman University, Mugla 48000, Türkiye; onderyildirim@mu.edu.tr; 4 Department of Veterinary Medicine, Devrekani TOBB Vocational School, University of Kastamonu, Kastamonu 37200, Türkiye; ertugrulterzi@gmail.com; 5 Department of Marine Biology and Environmental Science, College of Science and Environment, Mindanao State University Naawan, Naawan 9023, Misamis Oriental, Philippines; ruby.gonzales@msunaawan.edu.ph; 6 Department of Fisheries, Faculty of Fisheries, Mindanao State University Naawan, Naawan 9023, Misamis Oriental, Philippines; danarriesgado@yahoo.com (D.M.A.); victor.navarro@msunaawan.edu.ph (V.R.N.); 7 Department of Pharmacology and Toxicology, Faculty of Veterinary Medicine, University of Selcuk, Konya 42031, Türkiye; kuney@selcuk.edu.tr

**Keywords:** bioavailability, ceftriaxone, muscle, Nile tilapia, pharmacokinetic

## Abstract

**Background/Objectives**: The aim of this study was to determine the plasma and muscle pharmacokinetics of ceftriaxone (25 mg/kg) in tilapia after different administration routes. **Methods**: Two hundred and sixteen fish maintained at 30 ± 1.5 °C were divided equally into three treatment groups: intravascular (IV), intraperitoneal (IP), and intramuscular (IM). Ceftriaxone concentrations were quantified using high-performance liquid chromatography, and pharmacokinetic parameters were calculated by non-compartmental analysis. **Results**: The plasma total body clearance, volume of distribution at steady state, and elimination half-life (t_1/2λz_) were 0.22 L/h/kg, 0.85 L/kg, and 5.27 h, respectively. The t_1/2λz_ values were comparable among the IV, IP, and IM injection groups. The peak plasma concentration was 37.71 ± 3.12 µg/mL and 40.51 ± 2.77 µg/mL following IP and IM injection, respectively. The bioavailability was 67.04% for IP and 101.48% for IM. The peak muscle concentration was 9.49 ± 0.75 µg/g for IV, 5.71 ± 0.85 µg/g for IP, and 12.24 ± 2.41 µg/g for IM injection. The AUC_0–∞muscle_/AUC_0–∞plasma_ ratio was 0.23, 0.18, and 0.30 for the IV, IP, and IM groups, respectively. The AUC_muscle_/AUC_plasma_ indicates the ratio of drug penetration into the muscle, and a value less than 1 indicates that ceftriaxone penetrates into muscle tissue at a low ratio. **Conclusions**: These results indicate that ceftriaxone is well absorbed after IP and IM injections and passes into muscle tissue at a low tissue penetration. Ceftriaxone can be administered via IP and IM injection in Nile tilapia; nevertheless, its therapeutic efficacy requires evaluation.

## 1. Introduction

Nile tilapia, which is farmed in 83 countries around the world, reached nearly 5.2 million tons in 2023, generating a value of 11 billion dollars [1]. The tilapia stock available is characterized by rapid growth, disease resistance, and resilience to climate change [2]. However, stock density, poor environmental conditions, and stress-related bacterial infections that have become widespread due to increased tilapia farming have become a threat affecting both the fish supply and farmers’ livelihoods [3]. Therefore, antibiotics are commonly utilized in aquaculture for the treatment of diseases and prevention [3]. Licensed antibiotics in aquaculture are limited, and widespread and inappropriate use of these antibiotics leads to the emergence of bacterial resistance [4]. Because bacterial resistance limits antibiotic use, new antibiotic choices are required [5].

Ceftriaxone (CTX) is a parenteral third-generation cephalosporin [6]. The bactericidal action of CTX is due to its inhibition of bacterial cell wall synthesis [7]. It is a notable antibiotic known for its strong antibacterial efficacy, broad spectrum of activity, and low adverse effects [8]. It is effective against a wide range of bacteria, including both Gram-negative and Gram-positive species, as well as anaerobic strains [6]. It is resistant to hydrolysis by various beta-lactamases [7]. CTX is effective in treating infections of the skin and soft tissue, bones and joints, abdomen, urinary tract, lower respiratory tract, meninges, and pelvic area caused by susceptible organisms in humans [9]. The European Medicines Agency (EMA) has classified third-generation cephalosporins as category B (restricted) antibiotics for veterinary use due to the risks of antimicrobial resistance in animals and public health. It is recommended for treating clinical conditions that do not have effective alternatives in a lower category [10]. Although CTX is not approved for use in animals by the EMA or the Food and Drug Administration, it is approved for use in farm and pet animals for these infections in other countries, such as China and Bangladesh [11,12]. The bacterial resistance development against antibiotics such as tetracycline and fluoroquinolone, commonly used in fish, has been reported [13]. CTX is not approved for fish, but its superior pharmacokinetic and pharmacodynamic properties make it a good alternative antibiotic in fish [5].

Parenteral antibiotic administration is not common in fish due to some significant disadvantages [14,15]. However, parenteral administration has the advantages of high bioavailability, rapid onset of action, precise dosing, and lower drug use, and has been shown to be more effective than oral administration [16,17]. Recently, parenteral antibiotic use in fish has been found to be applicable in Korean culture farms [14]. CTX is a parenteral antibiotic used in veterinary medicine; however, data about its administration in fish remains inadequate. The pharmacokinetics of CTX have been established only in brown trout, and its application is advised owing to its extended half-life [5]. Furthermore, it was determined that *Aeromonas* spp. isolated from fish exhibited sensitivity to this drug [18,19]. *Aeromonas* spp., *Vibrio* spp., and *Streptococcus* spp. bacteria have been identified as common infectious agents in tilapia [20]. CTX has a broad spectrum of activity covering these bacteria [5]. Fish are heterothermic organisms, and changes in water temperature can modify their physiology and, subsequently, their pharmacokinetics. Therefore, pharmacokinetic studies are crucial in determining the appropriate dosage regimen of the drug in any fish species [21]. Considering this information, pharmacokinetic data obtained from trout, a cold-water species, are inappropriate for use with tilapia, a warm-water species [22]. To our knowledge, there is no information on the plasma and muscle pharmacokinetics of CTX in tilapia. This study aimed to determine the plasma and muscle pharmacokinetics of CTX after intravascular (IV), intraperitoneal (IP), and intramuscular (IM) administration in Nile tilapia at a dose of 25 mg/kg.

## 2. Results

### 2.1. HPLC Method

The retention time of CTX for plasma and muscle was 8.5 min with a total run time of 12 min. The endogenous matrix from plasma and muscle did not interfere with the peak of CTX. The calibration curve varied between 0.05 μg/mL and 100 μg/mL for plasma and 0.05 μg/g and 20 μg/g for muscle, was reproducible over the concentration range studied, and showed linearity with a regression coefficient (R^2^) > 0.995. For plasma and muscle, the limits of detection (LOD) and limits of quantitation (LOQ) were 0.025 and 0.05 μg/mL (g), respectively, based on a signal-to-noise ratio >3 and >6. The recovery of CTX for plasma and muscle was >91% to >87%, respectively. The intra- and inter-day coefficients of variation ranged from 3.45 to 6.04%. The intra- and inter-day bias of CTX ranged from −1.32 to 3.16%.

### 2.2. Animals

The IV, IP, and IM administration of ceftriaxone at a dose of 25 mg/kg to tilapia was generally well tolerated. No differences were observed in the swimming behavior of the fish during the study.

### 2.3. Plasma Pharmacokinetic Parameters

**Figure 1:**
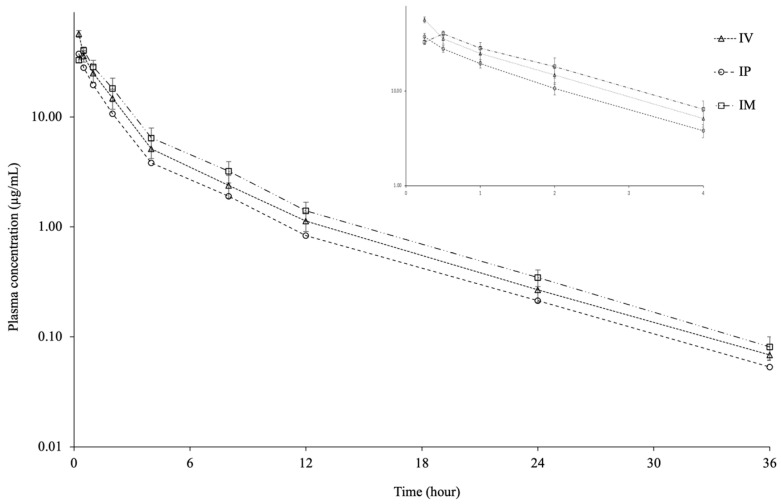
Semi-logarithmic plasma concentration-time curves following intravascular (IV), intraperitoneal (IP), and intramuscular (IM) administrations of ceftriaxone at a single dose of 25 mg/kg in Nile tilapia (*Oreochromis niloticus*) at 30 ± 1.5 °C (mean ± SD, *n* = 6).

Figure 1 shows the plasma CTX concentration–time curve following a single dose IV, IP, and IM injection. CTX was detected in plasma for up to 36 h after IV, IP, and IM injection. The pharmacokinetic parameters of CTX following a single dose IV, IP, and IM injection are shown in Table 1. The C_max_ and T_max_ of CTX were 37.71 ± 3.12 µg/mL and 0.25 h for IP injection and 40.51 ± 2.77 µg/mL and 0.50 h for IM injection. The C_0.25h_ after IV injection was 57.37 ± 4.01 µg/mL. The t_1/2λz_ of 5.27 h, V_darea_ of 1.67 L/kg, V_dss_ of 0.85 L/kg, and Cl_T_ of 0.22 L/h/kg were calculated following IV injection, respectively. The t_1/2λz_ values were similar in the IV, IP, and IM injection groups. The AUC_0–36_ was similar in the IV and IM injection groups but lower in the IP group. The bioavailability of CTX was 67.04% for IP injection and 101.48% for IM injection.

### 2.4. Muscle Pharmacokinetic Parameters

**Figure 2:**
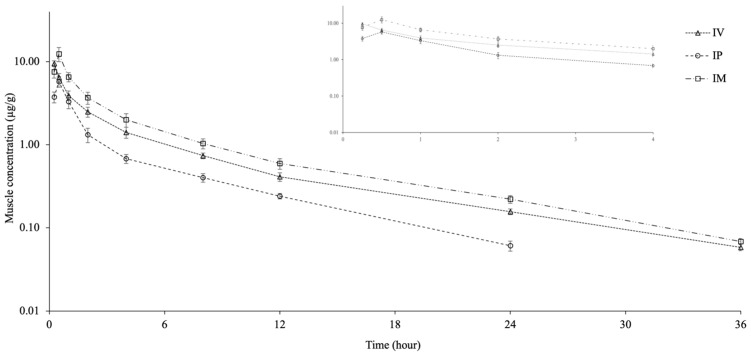
Semi-logarithmic muscle concentration-time curves following intravascular (IV), intraperitoneal (IP), and intramuscular (IM) administrations of ceftriaxone at a single dose of 25 mg/kg in Nile tilapia (*Oreochromis niloticus*) at 30 ± 1.5 °C (mean ± SD, *n* = 6).

The muscle concentration-time curves and the pharmacokinetic parameters for CTX following a single dose IV, IP, and IM injection are shown in Figure 2 and Table 2. CTX was detected in muscle for up to 36 h after IV and IM injection and for up to 24 h after IP injection. The C_max_ and T_max_ of CTX were 9.49 ± 0.75 µg/g and 0.25 h for IV injection, 5.71 ± 0.85 µg/g and 0.50 h for IP injection, and 12.24 ± 2.41 µg/g and 0.50 h for IM injection. The t_1/2λz_ was similar in the IV and IM groups but shorter in the IP group. The AUC_last_ was obtained in the order IM > IV > IP and was different between groups. The AUC_0–∞muscle_/AUC_0–∞plasma_ ratio was 0.23, 0.18, and 0.30 for the IV, IP, and IM groups, respectively.

## 3. Discussion

Injection of antibiotics may be preferred for valuable fish such as brood stock and aquarium fish and for fish such as olive flounders, which are generally calm and do not undergo excessive stress during injection [5,13]. In recent years, studies on the pharmacokinetics of injectable cephalosporin antibiotics in fish such as tilapia and trout have increased [5,23,24,25,26]. CTX used in veterinary medicine and appropriate for parenteral administration, is also applicable to Nile tilapia owing to its broad spectrum of activity and low adverse effects. This study demonstrates the pharmacokinetics of CTX in Nile tilapia for the first time, and this information may contribute significantly to the use of this drug in this species.

No side effects were observed after IV, IP, and IM injection of CTX at a dose of 25 mg/kg in Nile tilapia. No local or systemic adverse effects were noted following IM and IV administration of 25 mg/kg of brown trout [5]. The CTX dose of 25 mg/kg was determined considering previous studies in fish [5] and crocodiles [27].

The t_1/2λz_ following IV injection of CTX at a dose of 25 mg/kg in Nile tilapia at 30 ± 1.5 °C was 5.27 h. This value was shorter than the t_1/2λz_ value (18.22 h) obtained in brown trout (10–13 °C) at the same dose and administration route [5]. Fish are heterothermic organisms, and variations in water temperature influence their physiology and metabolism [28]. Physiological and metabolic differences among fish species may alter the V_d_ and Cl_T_ values of drugs [21]. The t_1/2λz_ is a hybrid parameter formed by Cl_T_ and V_d_ [29], and the fact that t_1/2λz_ of CTX is so variable between the two fish species may be due to the difference in these parameters.

V_darea_ is associated with body clearance, whereas V_dss_ is not influenced by clearance. In Nile tilapia, the V_darea_ of CTX (1.67 L/kg) was double that of V_dss_ (0.85 L/kg). Generally, V_darea_ is higher than V_dss_, but a large difference indicates that a large portion of the drug is eliminated before reaching pseudo-equilibrium [30]. CTX has a hydrophilic nature and a generally low V_dss_ (0.16–0.28 L/kg) in mammals [31,32]. However, it showed a wide V_dss_ in foals and calves [33,34]. Following IV injection in Nile tilapia, CFX showed a wide V_dss_ of 0.85 L/kg, indicating extensive penetration into various body tissues and fluids. Plasma protein binding ratio and body composition influence the V_dss_ [35]. The plasma protein binding ratio of CTX in fish is unknown, but binding to 29–45% has been reported in mammals [36,37]. In fish, plasma protein concentrations and acidic drug binding are lower than in mammals [38,39]. The V_dss_ may be wide in tilapia due to differences in body components and plasma protein binding. However, the V_dss_ value in Nile tilapia (0.85 L/kg, 30 ± 1.5 °C) was approximately 10 times higher than that in brown trout (0.09 L/kg, 10–13 °C) [5]. The water temperature influences various physiological parameters in fish, including tissue perfusion, cardiac output, and blood acid–base equilibrium [40,41]. The difference in V_dss_ between these two fish species may be due to physiological changes depending on water temperature and differences in the binding ratio to body components and plasma proteins.

There is no information on the biotransformation and excretion pathways of CTX in fish. The metabolism of CTX varies among species. While it does not undergo systemic biotransformation in humans, its metabolite has been detected in goats [42,43]. It is eliminated from the body through bile and urine [44]. The Cl_T_ of CTX following IV injection in Nile tilapia was 0.22 L/h/kg; this value is approximately 10 times higher than reported (0.02 L/h/kg) in brown trout [5]. Fish have biotransformation enzymes, but their content varies depending on the species, and the kidneys, bile, and gills play a role in the excretion of drugs from the body [38,45,46]. In fish, low ambient water temperature causes slow metabolic rate, biotransformation, and excretion of drugs [21,47]. The fast Cl_T_ of CTX in Nile tilapia may be due to rapid metabolism and excretion due to high water temperature.

CTX was well absorbed after IP and IM injection, exhibiting 67.04% and 101.48% bioavailability, respectively. There is no information on the IP bioavailability of CTX. The IM bioavailability was consistent with that previously reported (84–102%) in mammals [36,48]. It is recommended that a drug have >30% bioavailability to be useful in aquaculture [49]. CTX can be administered to Nile tilapia via IM or IP injection due to its high bioavailability. The IM bioavailability of CTX was very low (27.19%) in brown trout [5]. Water temperature variations influenced the oral bioavailability of enrofloxacin in trout. The oral bioavailability of enrofloxacin was 25% and 43% at 10 and 15 °C water temperatures, respectively [49]. The effect of temperature on IM bioavailability in fish is unknown. However, the difference in IM bioavailability in Nile tilapia and trout may be due to changes in cardiac output and tissue perfusion rate depending on temperature. The bioavailability after extravascular injection is generally less than or equal to 100% [50]. The bioavailability exceeding 100% may result from several errors, including mechanical (e.g., employing different animals in treatment groups), experimental (e.g., during sample collection, preparation, and storage), and analytical technique errors [50].

The C_max_ of CTX at a 25 mg/kg dose in Nile tilapia was 37.71 ± 3.12 µg/mL at 0.25 h for IP injection and 40.51 ± 2.77 µg/mL at 0.5 h for IM injections. These results showed that C_max_ was similar after IP and IM injection. The C_max_ obtained following IM injection of 25 mg/kg in Nile tilapia was lower than the value previously recorded (87.92 µg/mL) in brown trout [5]. This difference in C_max_ may be due to differences in drug formulation, Cl_T_, V_d_, and bioavailability.

The t_1/2λz_ value obtained in muscle tissue after IV and IM injection was longer than plasma. Similar results have been observed in brown trout [5]. The C_max_ was obtained in the order IM > IV > IP and was different between groups. The AUC_tissue_/AUC_plasma_ indicates the ratio of drug penetration into the tissue, and a value >1 indicates that it penetrates into the tissue at a higher ratio than plasma. The tissue penetration ratio of CTX is generally low. The AUC_0–∞muscle_/AUC_0–∞plasma_ ratio in the IV, IP, and IM groups was 0.23, 0.18, and 0.30, respectively, indicating low muscle penetration of CTX. This value was 0.02 and 0.04 for IV and IM applications in brown trout, respectively [5]. These results indicate that CTX penetration into muscle tissue is approximately 10 times greater in Nile tilapia than in brown trout. The difference in muscle tissue transition between the two fish species may be due to changes in tissue blood flow depending on water temperature.

The amount of drug in edible tissues of fish above the maximum residue limits (MRLs) causes undesirable effects and the development of bacterial resistance [21]. Therefore, it is very important to pay attention to the MRL value in edible tissues. To our knowledge, the MRL value of CTX has not been determined in food-valued animals. Muscle concentration of CTX decreased below the LOQ value (0.05 µg/g) at 36, 24, and 36 h after IV, IP, and IM injection, respectively. However, to determine the withdrawal time in fish, regulatory bodies responsible for the protection of human health need to determine the MRL values for CTX in muscle tissue, and tissue depletion studies need to be conducted [5].

Beta-lactams are a time-dependent class of antibiotics, with drug concentration having little effect on the overall rate and amount of bacterial death. Instead, it is critical to keep the free drug concentration above the organism’s MIC for a portion of the dosing period [51]. For successful beta-lactam therapy and to prevent the development of resistance, the percentage of a drug that remains above the MIC throughout the dosing interval to provide maximum bactericidal effect (T > MIC) is crucial. For therapeutic efficacy, a T > MIC value is desired to be >50% [52]. The MIC value of CTX for bacteria isolated from fish has not been determined. Therefore, the evaluation was made based on the susceptible breakpoint MIC values reported for CTX by CLSI for *Enterobacteriaceae* spp. (1 µg/mL) and *Streptococcus* spp. (0.5 µg/mL) [53]. Because plasma protein binding was not determined in this study, free plasma concentration was calculated by averaging plasma protein binding ratios previously reported (29–45%) in mammals [36,37]. The T > MIC 50% value provided 24 h for bacteria with MIC of 0.5 µg/mL and 16 h for bacteria with MIC of 1 µg/mL across all administration routes.

This research acknowledges several limitations. Critically, the absence of data regarding CTX’s plasma protein binding ratio, metabolic pathways, and excretion routes represents a significant gap in understanding its systemic disposition. Furthermore, the study’s failure to establish the MIC and demonstrate therapeutic efficacy against susceptible bacterial strains limits the translational applicability of the findings. The use of healthy animals, rather than those with induced or naturally occurring infections, further restricts the assessment of CTX’s clinical relevance. The study also lacks pharmacokinetic data following multiple-dose administrations, which is crucial for determining optimal dosing regimens and predicting potential accumulation effects. Importantly, the influence of water temperature on CTX’s pharmacokinetic profile was not investigated. Water temperature can significantly impact drug stability, absorption, and distribution, potentially altering its bioavailability and therapeutic efficacy. Moreover, the investigation did not include an assessment of residue levels and withdrawal time in tissues. Determining tissue residue levels is essential for assessing potential human exposure and ensuring compliance with regulatory limits.

## 4. Materials and Methods

### 4.1. Chemicals

The CTX analytical standard was purchased from Sigma-Aldrich (St. Louis, MO, USA). HPLC-grade methanol was brought from J.T. Baker (Gliwice, Poland). Trifluoroacetic acid was obtained from Merck (Darmstadt, Germany). The injectable formulation of CTX (Forsef, 1000 mg, Bilim Pharmaceuticals, Istanbul, Türkiye) was used for drug administration to fish.

### 4.2. Animals

The Institutional Animal Care and Use Committee at Mindanao State University granted ethical approval for the study (approval no: 2019/05). The experimental procedures were conducted at the Naawan campus, located in Misamis Oriental, Philippines. Nile tilapia (*Oreochromis niloticus*) exhibiting no external injuries or signs of disease, and displaying normal morphological and behavioral characteristics, were classified as clinically healthy. A total of 216 such individuals, each weighing between 100 and 120 g, were utilized in the study. These fish were randomly distributed across eight plastic fiberglass tanks, each with a volume of 400 L and housing 27 fish. To adjust to the environment, the fish endured 10 days in their tanks before the experiments. Throughout the experimental period, the water temperature was kept at 30 ± 1.5 °C, and a natural light/dark cycle was maintained using ambient daylight. The fish were fed with the commercial feed (Tilapia Grower, Tateh Aquafeeds, Quezon City, Philippines) twice a day until they were satiated.

### 4.3. Experimental Design

Two hundred and sixteen fish were randomly divided into three treatment groups: IV, IP, and IM, each including seventy-two animals. Each group (*n* = 72) was randomly divided into twelve subgroups of six fish, and one subgroup (*n* = 6) was used at each sampling time. IV, IP, and IM injections were performed from the caudal vessel, peritoneal cavity, and right epaxial muscle, respectively. CTX was administered at a dose of 25 mg/kg in all three administration groups. For drug administration to fish, the commercial formulation of CTX was diluted to 20 mg/mL with sterile water immediately before drug administration. Therefore, the fish received doses of CTX ranging from 0.125 to 0.150 mL based on their weight. Drug administration and blood collection were performed with an insulin syringe (1 mL, 26-gauge 1/2-inch needle) under tricaine methanesulfonate anesthesia (MS-222, 150 mg/L). Blood samples (1 mL) were taken from the caudal vessel at 0 (control), 0.25, 0.5, 1, 2, 4, 8, 12, 24, 36, 48, and 72 h. Following blood collection, the fish were promptly euthanized through immersion in a high dose of MS-222 (250 mg/L) solution, after which muscle (left epaxial muscle) samples were obtained at each specified time point. Blood samples were immediately placed in tubes containing lithium heparin, mixed by inversion multiple times, and stored in cold boxes. Blood samples were centrifuged (4000× *g* for 10 min) within one hour, and plasma samples were obtained. Plasma and muscle tissues were kept at −80 °C until CTX analysis.

### 4.4. Ceftriaxone Analysis

CTX analysis from plasma and muscle samples was performed by HPLC using the previously mentioned method [5]. The HPLC system (Shimadzu, Tokyo, Japan) was equipped with a model SPD-20A UV detector, a model SIL 20A auto-sampler, a model LC-20AT pump, a model DGU-20A degasser, and a model CTO-10A column oven. The chromatographic separation of CTX from plasma and muscle samples was carried out at 274 nm wavelength and using a C18 column (4.6 × 250 mm^2^; 5 μm, GL Sciences, Tokyo, Japan). The column temperature was kept at 35 °C via a column oven. The mobile phase consisted of methanol (35%) and trifluoroacetic acid (0.1% in ultrapure water) with a flow rate of 1 mL/min. The analysis of data was performed with Shimadzu Corp.’s LC Solution software (Version 1.25 SP5). The sample was thawed at room temperature for plasma and muscle analyses. One gram of muscle tissue was weighed, transferred to homogenization tubes, and homogenized. One hundred μL of plasma and 100 mg of muscle tissue were transferred to 2 mL microcentrifuge tubes, and 200 μL of methanol was added. The samples were vortexed for 30 s and then centrifuged at 10,000× *g* for 10 min. The supernatant was transferred to autosampler vials, and 20 μL was injected into the HPLC system.

### 4.5. Pharmacokinetic Analysis

Since repeated blood collection from fish was not possible (blood volume limitations), different fish were used at each sampling time, and CTX concentrations in plasma and muscle tissue were calculated for six animals in each group. However, as distinct animals were used at each sampling time, pharmacokinetic calculations were not performed individually for each animal but were instead based on the mean concentrations of six animals at each sampling point [22,54]. The use of curves that only show the mean may obscure the variability in concentrations between animals. Plasma and muscle concentrations of CTX were reported as mean ± standard deviation (SD).

The plasma and muscle CTX concentration versus time profile from fish in the IV, IP, and IM groups was subjected to pharmacokinetic analysis using Winnonlin software version 6.1.0.173 (Pharsight Corp., Mountain View, CA, USA). Pharmacokinetic data were calculated by non-compartmental analysis. The following pharmacokinetic parameters were determined for plasma: peak concentration (C_max_), plasma concentration at the first sampling time (0.25 h) after IV injection (C0.25 h), time to reach C_max_ (T_max_), area under the concentration-versus time curve from 0 to last h and 0 to infinity (AUC_0–last_, AUC_0–∞_), area under the plasma concentration–time curve extrapolated from tlast to ∞ in % of the total AUC (AUC_extrap%_), terminal elimination half-life (t_1/2λz_), apparent volume of distribution (V_d_), volume of distribution at steady state (V_dss_), total body clearance (Cl_T_), and mean residence time (MRT). The following parameters were calculated for the muscle: C_max_, T_max_, AUC, AUC_extrap%,_ and t_1/2λz_. The AUC was calculated using the linear-log trapezoidal method for IV application and the linear-up log-down method for IP and IM application. The C_max_, C_0.25h_, and T_max_ were determined from the individual plasma and muscle concentration–time profiles. The bioavailability (F) was determined using (AUC_IP,IM_/AUC_IV_) × 100.

## 5. Conclusions

This study presents the plasma and muscle pharmacokinetics of CTX in Nile tilapia for the first time. CTX demonstrates rapid absorption and substantial systemic exposure following IP and IM administration at a 25 mg/kg dose. CTX exhibited larger V_d_ and shorter t_1/2λz_ compared with cold-water species such as brown trout, with Cl_T_ reflecting temperature-dependent metabolic and excretory processes. Bioavailability was good after IP and IM injection, while muscle penetration remained limited (AUC_muscle/plasma_ ratios of 0.18–0.30), indicating low tissue accumulation similar to other cephalosporins. Overall, CTX was determined to be pharmacokinetically appropriate for parenteral use in tilapia. However, further research addressing the identified limitations is necessary to fully elucidate its therapeutic utility and ensure its safe and effective use in this economically important fish species.

## Figures and Tables

**Table 1:** Plasma pharmacokinetic parameters following intravascular (IV), intraperitoneal (IP), and intramuscular (IM) administrations of ceftriaxone at a single dose of 25 mg/kg in Nile tilapia (*Oreochromis niloticus*) at 30 ± 1.5 °C (*n* = 6).

Parameter	IV	IP	IM
t_1/2λz_ (h)	5.27	5.61	5.44
AUC_0–36_ (h * µg/mL)	113.05	75.79	114.72
AUC_0–∞_ (h * µg/mL)	113.57	76.22	115.36
AUC_extrap_ (%)	0.46	0.56	0.55
MRT_0–∞_ (h)	3.85	4.40	4.75
Cl_T_ (L/h/kg)	0.22	-	-
V_darea_ (L/kg)	1.67	-	-
V_dss_ (L/kg)	0.85	-	-
C_max_ (µg/mL)	-	37.71 ± 3.12	40.51 ± 2.77
C_0.25h_ (µg/mL)	57.37 ± 4.01	-	-
T_max_ (h)	-	0.25	0.50
F (%)	-	67.04	101.48

t_1/2λz_; terminal elimination half-life, AUC; area under the concentration-versus time curve, AUC_extrap%_; area under the plasma concentration–time curve extrapolated from t_last_ to ∞ in % of the total AUC, MRT_0–∞_; mean residence time, Cl_T_; total body clearance, V_darea_; apparent volume of distribution, V_dss_; volume of distribution at steady state, C_max_; peak plasma concentration, C_0.25h_; plasma concentration at the first sampling time (0.25 h) after IV injection, T_max_; time to reach peak plasma concentration, F; bioavailability.

**Table 2:** Muscle tissue pharmacokinetic parameters following intravascular (IV), intraperitoneal (IP), and intramuscular (IM) administrations of ceftriaxone at a single dose of 25 mg/kg in Nile tilapia (*Oreochromis niloticus*) at 30 ± 1.5 °C (*n* = 6).

Parameter	IV	IP	IM
t_1/2λz_ (h)	7.27	5.79	7.36
AUC_0–last_ (h * µg/g)	25.13	12.88	33.63
AUC_0–∞_ (h * µg/g)	25.74	13.39	34.36
AUC_extrap_ (%)	2.38	3.80	2.11
C_max_ (µg/g)	9.49 ± 0.75	5.71 ± 0.85	12.24 ± 2.41
T_max_ (h)	0.25	0.50	0.50
AUC_0–∞muscle_/AUC_0–∞plasma_	0.23	0.18	0.30

t_1/2λz_; terminal elimination half-life, AUC; area under the concentration-versus time curve, AUC_extrap%_; area under the muscle concentration–time curve extrapolated from t_last_ to ∞ in % of the total AUC, C_max_; peak muscle concentration, T_max_; time to reach peak muscle concentration.

## Data Availability

The data presented in this study are available upon request from the corresponding author.
